# Spatiotemporal Distribution of Continuous Air Pollution and Its Relationship with Socioeconomic and Natural Factors in China

**DOI:** 10.3390/ijerph19116635

**Published:** 2022-05-29

**Authors:** Dongsheng Zhan, Qianyun Zhang, Xiaoren Xu, Chunshui Zeng

**Affiliations:** 1 School of Management, Zhejiang University of Technology, Hangzhou 310023, China; zhands@126.com (D.Z.); chennery@foxmail.com (Q.Z.); 2 Shandong Provincial Key Laboratory of Water and Soil Conservation and Environmental Protection, College of Resources and Environment, Linyi University, Linyi 276012, China; 3 College of Tourism, Fujian Normal University, Fuzhou 350117, China

**Keywords:** continuous air pollution, spatiotemporal distribution, driving factors, MWGR, China

## Abstract

Continuous air pollution (CAP) incidents last even longer and generate greater health hazards relative to conventional air pollution episodes. However, few studies have focused on the spatiotemporal distribution characteristics and driving factors of CAP in China. Drawing on the daily reported ground monitoring data on the ambient air quality in 2019 in China, this paper identifies the spatiotemporal distribution characteristics of CAP across 337 Chinese cities above the prefecture level using descriptive statistics and spatial statistical analysis methods, and further examines the spatial heterogeneity effects of both socioeconomic factors and natural factors on CAP with a Multiscale Geographically Weighted Regression (MGWR) model. The results show that the average proportion of CAP days in 2019 reached 11.50% of the whole year across Chinese cities, a figure equaling to about 65 days, while the average frequency, the maximum amount of days and the average amount of days of CAP were 8.02 times, 7.85 days and 4.20 days, respectively. Furthermore, there was a distinct spatiotemporal distribution disparity in CAP in China. Spatially, the areas with high proportions of CAP days were concentrated in the North China Plain and the Southwestern Xinjiang Autonomous Region in terms of the spatial pattern, while the proportion of CAP days showed a monthly W-shaped change in terms of the temporal pattern. In addition, the types of regions containing major pollutants during the CAP period could be divided into four types, including “Composite pollution”, “O_3_ + NO_2_ pollution”, “PM_10_ + PM_2.5_ pollution” and “O_3_ + PM_2.5_ pollution”, while the region type “PM_10_ + PM_2.5_ pollution” covered the highest number of cities. The MGWR model, characterized by multiple spatial scale impacts among the driving factors, outperformed the traditional OLS and GWR model, and both socioeconomic factors and natural factors were found to have a spatial non-stationary relationship with CAP in China. Our findings provide new policy insights for understanding the spatiotemporal distribution characteristics of CAP in urban China and can help the Chinese government make prevention and control measures of CAP incidents.

## 1. Introduction

China has achieved rapid socioeconomic development since the reform and opening up started in 1979. As the recent Chinese national statistics have shown [1], per capita GDP has increased from 367.87 billion yuan in 1978 to 99,086.51 billion yuan in 2019, and the urbanization level has also increased from 17.9% to 60.6% during this period. However, the past extensive urban development mode with a GDP-oriented goal has also damaged the ambient air quality in urban China, causing huge losses for urban sustainable development and people’s physical health [2,3]. According to the World Health Organization’s report, the annual amount of premature deaths caused by ambient air pollution in China has reached more than 1.2 million, accounting for about two-fifths of the total amount of premature deaths, and economic loss has reached approximately 1.4 trillion dollars each year. The World Bank’s report [4] also pointed out that about 87% of the population in the world is facing great threat due to air pollution incidents. Due to the frequent occurrences of air pollution, continuous air pollution (CAP) has also become a common incident in a large number of Chinese cities [5,6,7]. Emerging evidence has shown that CAP incidents are more detrimental to urban socioeconomic development and life expectancy than ordinary air pollution events [8,9,10]. Other studies documented that the occurrences of continuous air pollution are likely to increase the number of outpatient visits to the respiratory department, mental health department, etc. [11,12]. However, research on the spatiotemporal distribution characteristics of CAP and its driving factors is still lacking. Therefore, there is urgent need to conduct CAP studies for policy making on urban air pollution prevention and the construction of a healthy China across Chinese cities.

A large number of previous studies have explored the spatial and temporal distribution characteristics of air pollution concentrations. In terms of the spatial distribution of air pollution concentrations, numerous studies employed air pollution data interpreted by remote sensing to explore the spatial distribution characteristics of specific air pollutant concentrations at different spatial scales [13,14]. However, finer-scale air pollution studies using reported data from ground monitoring stations are still limited due to data availability, especially in developing countries such as China. Furthermore, although different types of air pollutants have been found with varying spatiotemporal distribution patterns, only a small number of studies have identified the spatiotemporal patterns of the Air Quality Index (AQI), which measures the comprehensive air pollution level by calculating the concentrations of multiple air pollutants [15,16]. In terms of the temporal distribution of air pollution, previous research primarily focused on inter-annual changes of air pollution concentrations using remote sensing data [17] but failed to capture the finer time scales such as the monthly or diurnal dynamics of continuous air pollution within a particular year. With the improvement of ground monitoring sites and the open sharing of air pollution data, recent emerging literature has utilized real-time monitoring data to analyze the temporal pattern of air pollutant concentrations on a finer time scale [18,19]. For instance, some scholars identified the temporal change characteristics of air pollutants across 12 months or daily, within 24 h, and found that air pollution levels in Chinese cities are higher in the winter and spring seasonally and are more serious in the morning and evening daily [3,20,21]. Although numerous studies have focused on the spatiotemporal distribution characteristics of air pollution, literature regarding the spatiotemporal distribution characteristics of CAP incidents is still lacking.

The driving factors of air pollutant concentrations have also been extensively studied. One strand of literature has found that socioeconomic factors are important determinants of the air pollution level [22,23], including population size, GDP per capita, urbanization level, foreign direct investment (FDI), road density, greening level, energy consumption and urban form, etc. Another strand of literature has found that natural factors such as temperature, precipitation, wind speed, air pressure, sunshine hours and relative humidity also have significant impacts on air pollutant concentrations [24,25]. Only a few studies have found that both socioeconomic factors and natural factors are associated with air pollutant concentrations [2,18,26]. However, there are still disputes on whether and to what degree the various driving factors contribute to air pollution levels in the empirical literature. For instance, some studies showed that population density, GDP per capita and FDI have positive impacts on air pollution levels [27,28], while other studies found that these identical factors are also negatively associated with air pollution levels [29,30]. This is partly due to the existence of a spatial heterogeneous relationship between the driving factors and air pollutant concentrations. That is, the effects of the driving factors on air pollutant concentrations tend to vary by location.

With respect to the research methods used in prior literature, multiple linear regression models and extended spatial econometric models are the most widely used methods to examine the determinants of air pollutant concentrations on a large spatial scale [27,31]. However, these frequently used methods are mostly based on global models, which can only identify the average effect of driving factors on air pollutant concentrations with a fixed parameter. Considering the potential existence of a spatially nonstationary relationship for the driving factors, more recent studies have used Geographically Weighted Regression (GWR) models to explore the spatial heterogeneity effects of the various impact factors on air pollution [32,33], but traditional GWR models assume a uniform spatial scale for all the focused driving factors, which is evidently contrary to reality. Therefore, air pollution studies using Geographically Weighted Regression models that consider multiscale effects are still very scarce.

To address these research gaps, this paper employs spatial statistical analysis and a Multi-scale Geographically Weighted Regression (MGWR) model to explore the spatiotemporal distribution characteristics and driving factors of CAP in China using daily air quality data from 2019 across 337 Chinese cities at a prefecture-level above. Specifically, this paper attempts to address the following research questions: (1) what are the spatial and temporal distribution characteristics of CAP in China; (2) what are the main driving factors of CAP in China; (3) whether there are spatial heterogeneous effects on CAP for both socioeconomic factors and natural factors; and (4) whether there is a spatial scale variation in the impact of the various driving factors.

## 2. Materials and Methods

### 2.1. Data Source

The ambient air quality data used in this study was collected from the daily reported data on the Air Quality Index in 2019 published by the Data Center of the Ministry of Ecology and Environment of China. The released open data on the air quality in urban China includes the Air Quality Index (AQI), the air pollutants’ concentration, the major pollutants, the grade of the air quality and the recorded date in the monitoring cities. Our study area was composed of 337 cities above the prefecture level. Since Laiwu City was merged into Jinan City in 2019, they were not taken into consideration in this study. Additionally, the counties directly governed in the Hubei and Hainan provinces along with the Special Administrative Regions such as Hong Kong, Macao and Taiwan province were not included in this study. The data on the socioeconomic factors came from the 2020 China City Statistical Yearbook and the local governments’ statistical bulletin, while the natural factors were obtained from the National Meteorological Information Centre (http://data.cma.cn/ (accessed on 2 March 2020)).

The Air Quality Index (AQI) is a dimensionless index that quantitatively describes the overall air quality of a specific city, and can indicate the overall air pollution level within the city [15]. The calculation formula of the AQI is as follows:

(1) $$\mathrm{AQI}\;=\;\mathrm{Max}\left({\mathrm{IAQI}}_{1},{\mathrm{IAQI}}_{2},{\mathrm{IAQI}}_{3},\;\ldots ,\;{\mathrm{IAQI}}_{n}\right)$$

where AQI is the Air Quality Index of a city; IAQI is the sub-index of the air quality of a city; *n* is the pollutants number, including sulfur dioxide (SO_2_), nitrogen dioxide (NO_2_), carbon monoxide (CO), ozone (O_3_), particulate matter (the particle diameter is less than or equal to 10, PM_10_) and particulate matter (the particle diameter is less than or equal to 2.5, PM_2.5_).

Table 1 lists the basic characteristics of the Air Quality Index (AQI). A lower Air Quality Index in a city suggests a better air quality in the city, while a higher Air Quality Index indicates a worse air quality in the city. When the daily AQI of a city is over 100, it indicates the presence of air pollution in the city. Major pollutants refer to the pollutants’ type of the maximum IAQI when the AQI exceeds 50 in a city.

### 2.2. Continuous Air Pollution Measurement

Referring to the existing research [34,35], continuous air pollution refers to the occurrence of air pollution (AQI > 100) in a city for at least three consecutive days, which can be documented as a continuous air pollution incident from the starting day to the end day during the CAP period. The measurement indicators of CAP in this study included the proportion of CAP days, the frequency of CAP, the maximum amount of CAP days and the average amount of CAP days.

### 2.3. Spatial Analysis Methods

#### 2.3.1. Bivariate Spatial Autocorrelation

The bivariate spatial autocorrelation method is an extended spatial autocorrelation analysis method, which is composed of bivariate global spatial autocorrelation and bivariate local spatial autocorrelation. Among them, the bivariate global spatial autocorrelation mainly examines the spatial correlation of the same attribute variable of the spatial object in different time periods or between two different attribute variables [36], while bivariate local spatial autocorrelation (Bivariate LISA) is used to explore the spatial association mode of the attribute value of the spatial unit in different time periods or the attribute value of the spatial research unit and the adjacent spatial unit in the local space. In this study, the bivariate global spatial autocorrelation method was used to measure the spatial correlation intensity between the proportion of CAP days and the proportion of regular air pollution days in Chinese cities, and the bivariate local spatial autocorrelation method was applied to analyze the spatial association pattern between the proportion of CAP days and the proportion of regular air pollution days in Chinese cities.

#### 2.3.2. Grouping Analysis

The grouping analysis tool is a newly developed spatial clustering analysis method embedded in ArcGIS 10.1 (ESRI, Redlands, CA, USA) and later software, combining both the feature attributes and spatial constraint characteristics of the research objects [37]. The number of spatial groups to be created should be given, which makes all the studied objects have the greatest attribute similarity in the intra-groups and the greatest difference in the inter-groups. The distinct advantage of the grouping analysis method is that the grouping results are spatially connected, which is more beneficial for urban planning and policy making. The effectiveness of grouping analysis can be measured by the Pseudo-F statistic, which is used to reflect the ratio of similarity within groups and the differences across groups. The calculation formula of the grouping analysis method is as follows:

(2) $$\mathrm{Pseudo}-F\;\mathrm{Statistics}\;=\frac{\left(\frac{R^{2}}{n_{c}-1}\right)}{\left(\frac{1-R^{2}}{n-n_{c}}\right)}$$

(3) $$R^{2}=\frac{\mathrm{SST}\;-\;\mathrm{SSE}}{\mathrm{SST}};\;\mathrm{SST}\;=\sum_{i=1}^{n_{c}}\sum_{j=1}^{n_{i}}\sum_{k=1}^{n_{v}}{\left(V_{ij-}^{k}V^{k}\right)}^{2};\;\mathrm{SSE}\;=\sum_{i=1}^{n_{c}}\sum_{j=1}^{n_{i}}\sum_{k=1}^{n_{v}}\left(V_{ij-}^{k}V_{i}^{k}\right)jk^{2}$$

where SST represents inter-groups variation, SSE represents the intra-group variation, *n* is the number of cities, $n_{i}$ is the number of cities in the group i, $n_{c}$ is the number of groups, $n_{v}$ is the number of variables used for the grouping analysis and $V_{ij}^{k}$ is the value of the variable k in the group i and the city j. $V^{k}$ refers to the mean of the variable k among all the Chinese cities and $V_{i}^{k}$ is the mean of the variable k in the group i.

#### 2.3.3. Multiscale Geographically Weighted Regression (MGWR)

Unlike the ordinary least squares (OLS) model, which is a globe regression with constant estimated parameters, Geographically Weighted Regression (GWR) is a local regression analysis that allows parameter estimates to differ across space [38]. However, Multiscale Geographically Weighted Regression is an extended and optimized model for the traditional Geographically Weighted Regression [39]. It allows the postulated local relationship between the explanatory variables and the dependent variable to change with different spatial scales in order to precisely capture the spatial scale range of the impacts. The calculation formula of MGWR is as follows:

(4) $$\log\left(Y_{i}\right)=\beta_{bw0}\left(u_{i},v_{i}\right)+\sum_{j}\beta_{bwj}\left(u_{i},v_{i}\right)\log X_{ij}+\varepsilon_{i}$$

where $\beta_{bw0}$ is the dependent variable; $\beta_{bwj}$ is the optimal bandwidth after the conditional relationship *j* is corrected; $X_{ij}$ is the explanatory variable *j*; and $\varepsilon_{i}$ is the residual term. Following previous literature [31,34], the explanatory variables in this study included both socioeconomic variables and natural variables. Specifically, the socioeconomic factors included the population density (popden), per capita GDP (pgdp), proportion of secondary industry added value in GDP (prosec), proportion of foreign direct investment in GDP (FDI), normalized difference vegetation index (NDVI), road density (roadden) and energy consumption (energy), while the natural factors included temperature (tem), precipitation (pre), wind speed (ws), relative humidity (rh), air pressure (ap) and sunshine duration (sd). MGWR 2.2.1 software (Arizona State University, Tempe, AZ, USA) was employed to run the model.

## 3. Results

### 3.1. Descriptive Statistical

In order to present the descriptive statistical characteristics of CAP in China, our study selected several interesting measurement indicators of CAP in the statistical analysis, including the proportion of CAP days, frequency of CAP, maximum amount of CAP days and the average amount of CAP days. Additionally, the proportion of air pollution days was also introduced as a reference. Table 2 shows the descriptive statistical results of the measurement indicators related to CAP across Chinese cities. It can be seen that in 2019, the average proportion of air pollution days across Chinese cities (AQI > 100) was 17.80%, which means that the average days with air pollution in the whole year over Chinese cities was about 65 days (days with air pollution = 17.80% × 365 = 65 days). The proportion of CAP days across Chinese cities varied from 0.00% to 73.15%, with a mean of 11.50%, indicating that the average proportion of CAP days accounted for 11.50% of the whole year in China. In other words, the average amount of CAP days was about 42 days in 2019 across Chinese cities (the average amount of CAP days = 11.50% × 365 = 42 days). Comparing the above two proportions, we can find that the proportion of CAP days across Chinese cities accounted for approximately 64.60% of the proportion of regular air pollution days, a figure indicating that about two-thirds of the regular air pollution days across Chinese cities were continuous air pollution. Thus, CAP as a more common air pollution incident in China should arouse widespread social attention.

As shown in Table 2, other measurement indicators of CAP show that the average frequency of CAP in China reached 8.02, the average maximum number of CAP days was 7.85 days and the average amount of CAP days ranged from 0 to 16.69 days with a mean of 4.20 days, suggesting that the average frequency of CAP was close to eight times, the maximum amount of CAP days was close to 8 days on average and the average length of CAP incidents was about 4 days. These findings show that CAP incidents occurred frequently and lasted much longer in 2019 across Chinese cities. Therefore, the public and Chinese government, especially the environmental protection departments, should pay great attention to CAP incidents.

### 3.2. Spatial Distribution Characteristics of CAP

**Figure 1:**
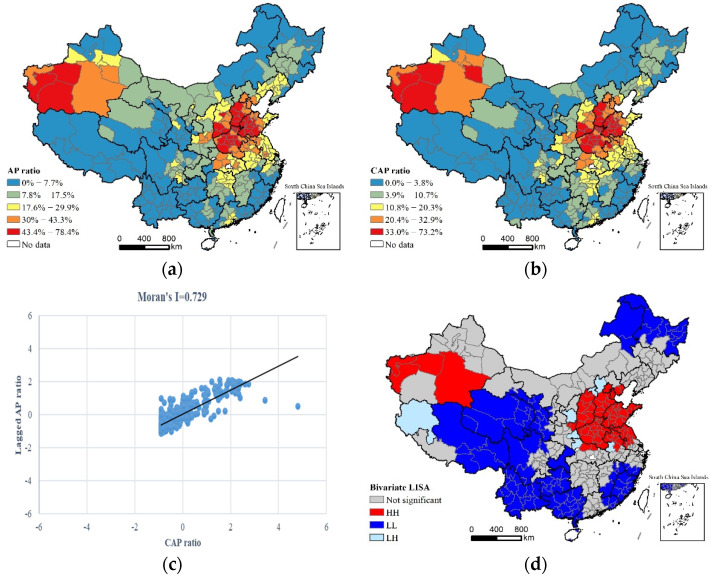
The spatial distribution and correlation characteristics between the proportion of CAP days and the proportion of air pollution days in China: (**a**) spatial distribution of proportion of air pollution days; (**b**) spatial distribution of proportion of CAP days; (**c**) scatter figure of Globe Moran’s I value for proportion of CAP days; and (**d**) bivariate LISA of proportion of CAP days and proportion of air pollution days.

Figure 1a,b shows the spatial distribution of the proportion of CAP days (abbreviated as the CAP ratio) and the proportion of air pollution days (abbreviated as the AP ratio) across Chinese cities, respectively. It can be found that the spatial distribution pattern between the proportion of CAP days and the proportion of air pollution days across Chinese cities was very similar. The high-value areas of the two measurement indicators were mainly concentrated in provinces such as the Henan, Shanxi, Shandong and Hebei provinces, municipalities such as Beijing and Tianjin, and several cities within the Xinjiang Autonomous Region, while the low-value areas of the two indicators were widely distributed in the provinces of Southwest China, the Southeastern coastal regions in China and Northeast China.

The bivariate spatial autocorrelation analysis method was used to analyze the spatial correlation intensity between the proportion of CAP days and the proportion of air pollution days across Chinese cities. As presented in Figure 1c, the bivariate globe spatial autocorrelation results showed that the bivariate Moran’s I index of the two observed indicators was 0.729 at the 0.01 significance level, indicating that the proportion of CAP days had a stronger spatial correlation intensity with the proportion of air pollution days across Chinese cities. Figure 1d further shows the spatial pattern of the bivariate local spatial autocorrelation. The HH type represents the high-value areas of both the proportion of CAP days and the proportion of air pollution days in China and covers a total of 79 cities, which were mainly distributed in cities in the North China Plain and some cities in the Xinjiang Autonomous Region. The LL type represents the low-value areas of both the proportion of CAP days and the proportion of air pollution days in China and includes a total of 107 cities, which were mainly distributed in several cities in the Southwest, Southeast and Northeast China. The LH type represents areas with a low proportion of CAP days across Chinese cities but the high proportion of air pollution days in the neighboring cities, including a total of seven cities such as Zhangjiakou, Datong, Shangluo, Shiyan, Lu’an, Yan’an and Ali. The HL type does not exist in our study, suggesting that an area with a high proportion of CAP days and a low proportion of air pollution days in the neighboring cities is not likely to happen.

**Figure 2:**
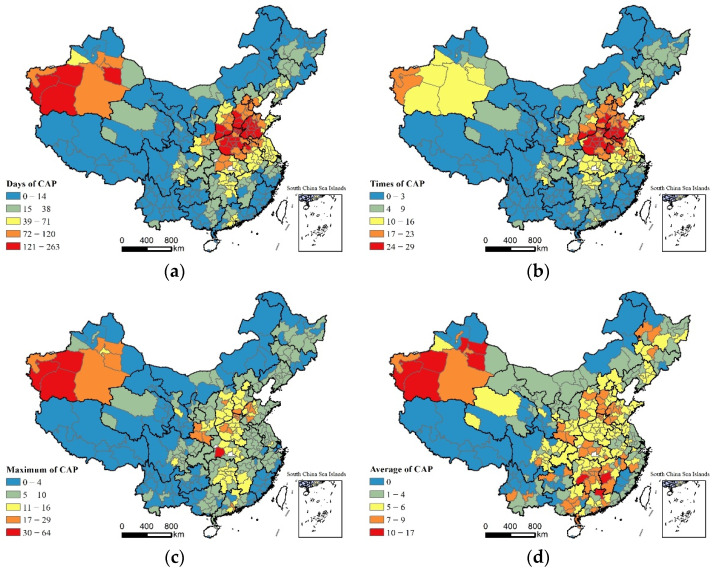
Spatial distribution of CAP-related measurement indicators in China: (**a**) spatial distribution of days of CAP; (**b**) spatial distribution of frequencies of CAP; (**c**) spatial distribution of maximum amount of CAP days; (**d**) spatial distribution of average amount of CAP days.

Figure 2 shows the spatial distribution of the CAP-related measurement indicators over Chinese cities. In terms of the spatial pattern for each indicator, areas with a greater number of CAP days (more than 72 days) were mainly concentrated in cities in the Bohai Sea Rim Region, Xinjiang Autonomous Region, etc. Areas with higher frequencies of CAP (more than 17 times) were also mainly distributed in the Bohai Rim Region and Xinjiang Autonomous Region. The areas with high average amount of CAP days were widely distributed, covering a large number of cities within the Northeast, North China, Southwest and Northwest regions. The areas with high amounts of maximum CAP days were concentrated in several districts within the Xinjiang Autonomous Region and a small number of cities within North China. This finding may be attributed to the fact that these areas typically observe more fossil energy consumption, a higher proportion of heavy industrial structure and many adverse natural conditions such as stronger winds and less precipitation.

### 3.3. Temporal Distribution Characteristics of CAP

**Figure 3:**
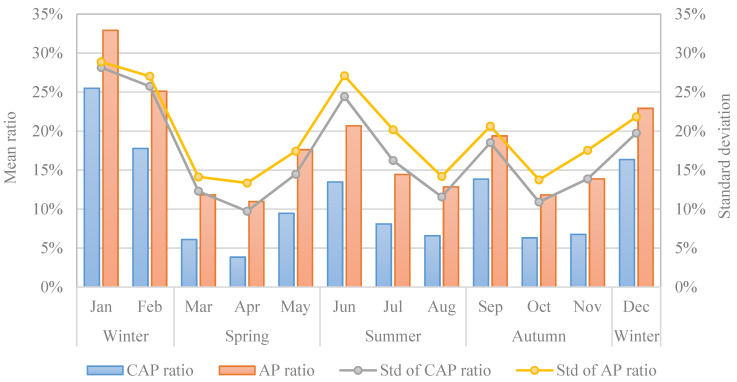
Temporal pattern of continuous air pollution in China.

Figure 3 presents the temporal distribution characteristics of the proportion of CAP days and the proportion of air pollution days across Chinese cities. As can be seen, the temporal variation of the proportion of CAP days and the proportion of air pollution days observed a high similarity, both showing W-typed change characteristics. Specifically, January, February, May, June, September and December witnessed higher values in both the proportion of CAP days and the proportion of air pollution days over Chinese cities. The correlation analysis result between the proportion of CAP days and the proportion of air pollution days in different months found that the Pearson correlation coefficient of the two indicators reached 0.993 and the *p* value passed the significance test at the 0.01 level, indicating that the presence of CAP incidents was highly correlated with regular air pollution episodes. Furthermore, the standard deviation of the two ratios was observed to increase with the mean CAP ratio and AP ratio, indicating that there was even a larger spatial variation in both the ordinary air pollution and CAP across Chinese cities in the heavily polluted months.

### 3.4. Region Types of the Major Pollutants during the CAP Periods

**Figure 4:**
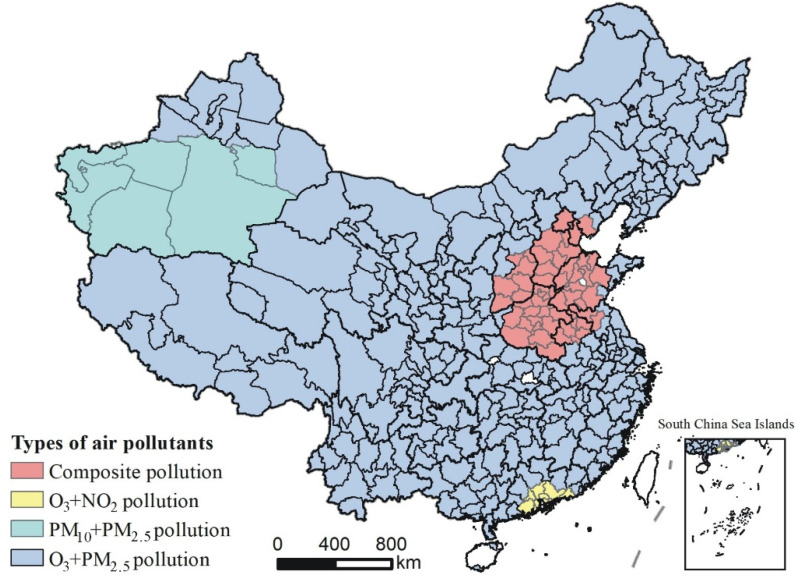
Types of regions of the major pollutants during the CAP period.

According to the occurrence frequency of the major pollutants during the CAP periods, we used the grouping analysis method in ArcGIS 10.8 to identify the types of regions that contained the major pollutants over all the Chinese cities. The Pseudo-F statistic results showed that the optimal grouping scheme was four groups, and the corresponding Pseudo-F statistic had the largest value of 139.81, indicating that the major pollutants’ region type during the CAP periods divided into four groups was the most reasonable. Table 3 lists the occurrence frequency of the major pollutants in each group, while Figure 4 maps the types of regions of the major pollutants during the CAP period. We can see that the first group was named “Composite Pollution”, with a total of 53 cities and diverse major pollutants, since the average occurrence frequencies of PM_10_, O_3_ and PM_2.5_ were 7.28 times, 58.17 times and 56.58 times, respectively, which all exceed their national mean. The second group was composed of nine cities in total and was named “O_3_ + NO_2_ pollution” due to the fact that the major pollutants with the highest frequencies were O_3_ and NO_2_, with an average occurrence frequency of 32.11 times and 3.11 times, respectively. The third group was “PM_10_ + PM_2.5_ pollution” and included six cities. The frequencies of the major pollutants in this group were relatively higher in PM_10_ and PM_2.5_, with an average of 130.33 times and 28.83 times, respectively. The fourth group was “O_3_ + PM_2.5_ pollution”, with a total of 269 cities. Although the average frequencies of all the kinds of major pollutants in the fourth group were still lower relative to the national mean, the average occurrence frequencies of the major pollutants were much higher in O_3_ and PM_2.5_ than the other pollutants, reaching 16.77 times and 20.49 times, respectively.

### 3.5. Spatial Heterogeneous Effects of the Driving Forces on CAP

#### 3.5.1. Goodness of Fit and Bandwidth

To explore the driving forces of CAP across Chinese cities at a local spatial scale, we employed a MGWR model to identify the spatial heterogeneity effects of CAP in China. Table 4 shows the compared results of the goodness of fit for the OLS, GWR and MGWR models. The residual sum of squares, AICc and adjusted R^2^ in the MGWR model were 35.712, 407.464 and 0.862, respectively. Except that the residual sum of squares of the MGWR model was slightly higher than that of the GWR model, the other fitting indicators were significantly better than those in the OLS model and the GWR model. On the whole, the MGWR model had an even better goodness of fit than the OLS and GWR models.

**Figure 5:**
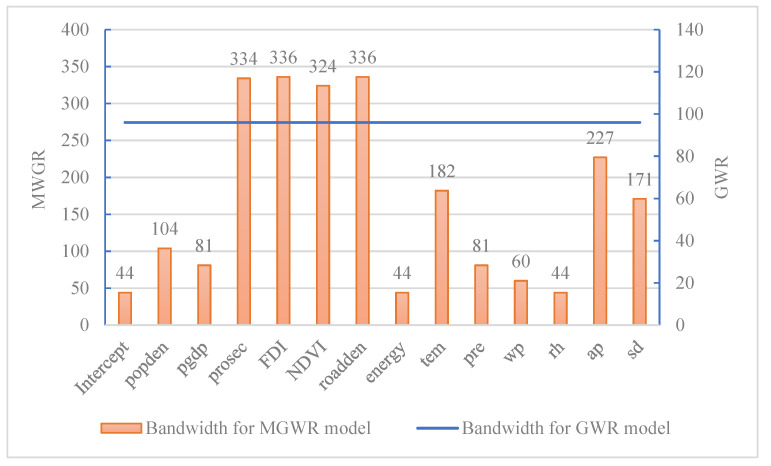
Bandwidth comparison between the GWR and MGWR models.

A bandwidth was used to measure the spatial impact range of the explanatory variables on CAP. Choosing the adaptive bisquare as the spatial kernel method and AICc as the optimal bandwidth selection criterion, the calculated bandwidth presented in Figure 5 showed that the bandwidth of the GWR model was 96, while the bandwidth of the MGWR model varied from 44 to 336. Among them, the explanatory variables such as the proportion of the secondary industry, the proportion of FDI and road density saw larger bandwidths that all exceeded 300, indicating that their impact range on the proportion of CAP days was close to the whole study area. Population density, temperature, air pressure and sunshine duration had the second largest bandwidth, with a bandwidth ranging from 104 to 227, indicating that their impact on the proportion of CAP days over Chinese cities was restricted to be regional. Per capita GDP, energy consumption, precipitation, wind speed, relative humidity and other explanatory variables had much smaller bandwidths ranging from 44 to 81, indicating that their impact on the proportion of CAP days over Chinese cities was local.

#### 3.5.2. Estimated Parameter Results

Table 5 shows the estimated parameter results of the OLS and MGWR models using the standardized data. The results of the OLS model showed that explanatory variables such as GDP per capita, NDVI, precipitation, wind speed, relative humidity and sunshine duration had a significant negative impact on the proportion of CAP days across Chinese cities, while the proportion of FDI, road density, temperature and air pressure had a significant positive effect on the proportion of CAP days. Unlike the results in the OLS model, the regression parameters of the MGWR model showed spatial heterogeneity effects. In terms of the estimated parameters in the MGWR model, variables such as population density, GDP per capita, energy consumption, temperature, precipitation, wind speed, relative humidity and sunshine duration had both positive and negative effects on CAP across Chinese cities. The proportion of FDI, NDVI and road density all had a negative impact on CAP while air pressure had a dominantly positive impact on CAP.

**Figure 6:**
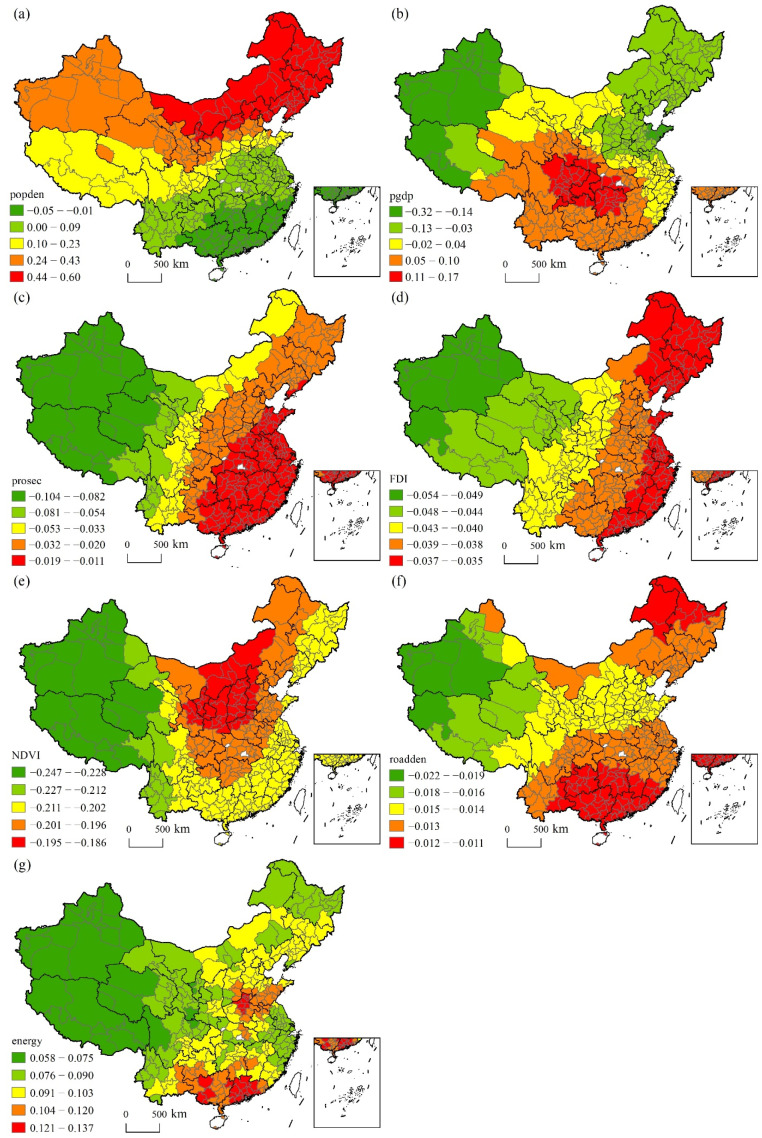
Spatial heterogeneous effects of socioeconomic factors: (**a**) population density, (**b**) per capita GDP, (**c**) proportion of secondary industry added value in GDP, (**d**) proportion of foreign direct investment in GDP, (**e**) NDVI, (**f**) road density and (**g**) energy consumption.

Figure 6 shows the spatial heterogeneous effects of the socioeconomic factors. Population density had a positive impact on CAP in most cities, with the impact intensity increasing from the south to the north, and was negatively associated with CAP only within a small part of Southeast China. GDP per capita had a positive impact on the occurrence of CAP in Central and Western China but had a negative impact in the Northwest, Northeast and North China Plains. The proportion of the secondary industry and FDI both had a negative impact on the occurrence of CAP, and the absolute value of its impact intensity was shown to increase from the east to the west. NDVI and road density both had a stronger inhibitory effect on CAP in the Western region, while energy consumption witnessed a greater impact on CAP in the middle and lower reaches of the Yellow River Basin and cities in South China.

**Figure 7:**
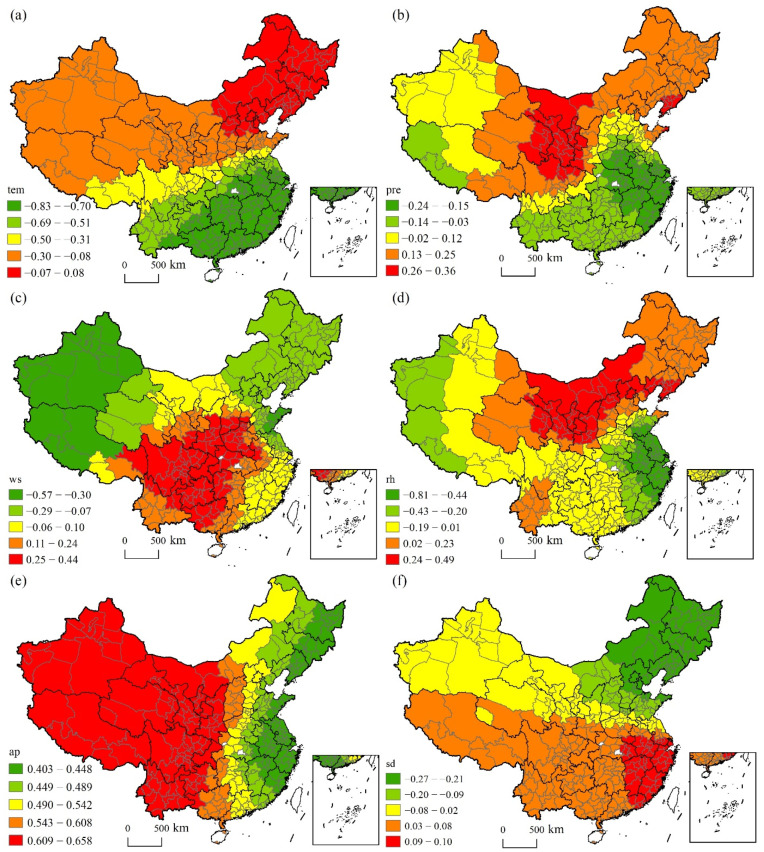
Spatial heterogeneous effects of natural factors: (**a**) temperature, (**b**) precipitation, (**c**) wind speed, (**d**) relative humidity, (**e**) air pressure and (**f**) sunshine duration.

Figure 7 shows the spatial heterogeneous effects of the natural factors. Temperature had a negative impact on CAP as expected, and its impact intensity showed an increasing trend from the north to the south. Precipitation had a good inhibitory effect on CAP in Southeast and Southwestern Tibet but contributed to CAP in the Northwest and Northeast regions. Wind speed witnessed a significant negative effect on CAP in the Northwest and Northeast regions but had a positive effect on CAP in the Central and Southwestern regions. Relative humidity had a negative impact on CAP in the Eastern coastal areas and the Western border areas but exerted a positive impact in the Northwest and Northeast regions and in several cities in the Yunnan province. Air pressure had a positive impact on CAP and observed an increasing impact intensity from the west to the east. Sunshine duration had a negative impact on CAP in Northeast China and small parts of North China but had a significant positive impact on most parts of the Southern region.

## 4. Discussion

Drawing on the daily reported data on the urban air quality in 2019 in China, this study first explored the spatiotemporal distribution characteristics of CAP incidents across 337 Chinese cities at the prefecture-level above and utilized a MGWR model to examine the spatially non-stationary relationship between socioeconomic factors coupled with natural factors and CAP occurrences. Our contributions adding to the literature include at least three aspects. First, this study adds empirical evidence of the spatiotemporal patterns of CAP incidents across Chinese cities on the national scale. Second, we used a MGWR model to reveal a multiscale spatial-varying relationship between the driving factors and CAP in China, outperforming the ordinary OLS and GWR models, which could provide a method reference for future air pollution studies. Third, our findings not only provide new insights for policy making on the environmental pollution governance in China, but also presents enlightenment for designing disease-prevention and health-promotion policies related to CAP incidents.

In line with previous air pollution studies in China [40,41], this study found that the proportion of CAP over Chinese cities presents a distinct spatial agglomeration characteristic, with the high-value areas mainly distributed in the North China Plain and Southwestern Xinjiang Autonomous Region. Its spatial pattern had a strong similarity and spatial correlation with that of the proportion of regular air pollution days. However, unlike other scholars who found that air pollution had a U-shaped change over the months [18,20,34], our study suggests that the proportion of CAP days across Chinese cities and the proportion of air pollution days both show a W-shaped change over the months. That is, the high proportion of CAP days is also likely to appear in the summer and autumn months such as June and September, suggesting that the recent air pollution control across Chinese cities has gradually changed from the past winter and spring seasons to full-time treatment in the four seasons.

According to the region types of the major pollutants during the CAP period, we found that the major pollutants’ region types across Chinese cities can be divided into four types as “Composite pollution”, “O_3_ + NO_2_ pollution”, “PM_10_ + PM_2.5_ pollution” and “O_3_ + PM_2.5_ pollution”, and the number of cities in each group is 53, 9, 6 and 269, respectively. This finding is similar to previous research on the region types of the major pollutants in China [2], indicating that “O_3_ + PM_2.5_ pollution” has the widest distribution in the whole of China.

Our study also indicated that a MGWR model with a varying spatial scale range has a better goodness of fit than the traditional OLS and GWR models. Both socioeconomic factors and natural factors had significant spatial heterogeneous effects on CAP over Chinese cities, but the average impact intensity of the natural factors was greater than that of the socioeconomic factors. In line with previous literature using GWR methods, the MGWR model results also showed that both the socioeconomic factors and natural factors witnessed a spatial non-stationary relationship with CAP over Chinese cities, verifying the existence of spatial heterogeneity effects in the impact direction and intensity of its driving factors.

Some limitations of our study should also be noted. First, this study used cross-sectional data, which fails to capture the dynamic change characteristics of CAP in each year and cannot reveal a causal relationship between driving factors and CAP. Future research should be concerned with the temporal evolution characteristics and driving factors of CAP through panel data. Second, due to the unavailability of the data, we have not identified the driving factors that affect CAP at a finer scale, such as the daily meteorological conditions. Last, this study only used a single AQI indicator in the city to define CAP incidents. Considering the fact that the spatiotemporal distribution characteristics and impact factors of CAP in different types of air pollutant indicators may be discrepant, future research should focus on CAP incidents with multiple air pollutant indicators such as PM_2.5_ and O_3_.

The findings in this paper have some important policy implications. First, the Chinese government and environmental management departments should attach great importance to CAP incidents to reduce their health hazards to urban residents. The North China Plain and the Southwestern region of the Xinjiang Autonomous Region are the current critical areas to abate CAP; thus, the investment of environmental pollution control should be increased in these important regions. Second, the government should pay attention to the more frequent CAP incidents in winter and some summer and autumn months. To abate the occurrence frequency of CAP incidents and their health threats, environmental management departments should strengthen monitoring and forecasting on CAP incidents to guide human travel behavior and people’s daily life activities from a time perspective. Finally, controlling and abating CAP needs to comply with local conditions. Due to the distinct spatiotemporal pattern and driving factors of CAP in different cities, the control measures of CAP should act according to the circumstances. On the whole, synergistic control of the ozone and PM_2.5_ pollutants should become a priority for most Chinese cities, and the government should combine the key driving factors of CAP in specific regions to carry out environmental pollution control and prevention actions.

## 5. Conclusions

Drawing on daily reported air quality data across 337 Chinese cities at the prefecture-level above, this paper used descriptive statistics and GIS spatial statistics analysis methods to explore the spatiotemporal distribution characteristics of CAP, and employed a MGWR model to identify a spatial non-stationary relationship between driving factors and CAP in China. Our study shows that CAP incidents were relatively serious in 2019 across 337 Chinese cities, with the average proportion of CAP days being 11.50% of the whole year and the average frequency of CAP and the maximum and average amount of CAP days over Chinese cities recorded as 8.02 times, 7.85 days and 4.20 days, respectively. With respect to the spatiotemporal pattern of CAP, the higher proportion of CAP days in China were mainly concentrated in the North China Plain and Southwestern Xinjiang Autonomous Region spatially, while CAP in China presented a W-shaped pattern over the months temporally. Moreover, the whole of China could be divided into four types of regions regarding the major pollutants during the CAP periods, including the region types of “Composite pollution”, “O_3_ + NO_2_ pollution”, “PM_10_ + PM_2.5_ pollution” and “O_3_ + PM_2.5_ pollution”, in which the region type of “O_3_ + PM_2.5_ pollution” was a dominant region type during the CAP periods. Last, the MGWR model witnessed a better goodness of fit in predicting the proposition of CAP days than the traditional OLS and GWR models, and both the socioeconomic factors and natural factors had multiscale spatial non-stationary impacts on CAP. For instance, the proportion of the secondary industries, FDI, NDVI and road density tended to have a negative impact; atmospheric pressure likely had a positive effect; and the other driving factors had both positive and negative effects on CAP across Chinese cities.

## Figures and Tables

**Table 1:** The basic characteristics of Air Quality Index.

Air Quality Index (AQI)	Air Quality Index Level	Air Quality Index Category	Major Pollutants
0–50	Level 1	Excellent	SO_2_, NO_2_, CO, O_3_, PM_10_, PM_2.5_
51–100	Level 2	Good	
101–150	Level 3	Light pollution	
151–200	Level 4	Moderate pollution	
201–300	Level 5	Heavy pollution	
>300	Level 6	Serious pollution	

**Table 2:** Descriptive statistics of CAP-related measurement indicators in China.

Measurement Indicators	Mean	Std. Dev.	Min	Max
Proportion of air pollution days (%)	17.80	16	0.00	78.36
Proportion of CAP days (%)	11.50	13	0.00	73.15
Frequency of CAP (times)	8.02	8.06	0.00	29.00
Maximum of CAP days (days)	7.85	6.96	0.00	64.00
Average of CAP days (days)	4.20	2.41	0.00	16.69

**Table 3:** Types of regions of the major pollutants during the CAP periods.

Types of Regions	PM_10_	O_3_	PM_2.5_	NO_2_	Count	Types
Group 1	7.28	58.17	56.58	0.06	53	Composite pollution
Group 2	0.00	32.11	0.22	3.11	9	O_3_ + NO_2_ pollution
Group 3	130.33	0.67	28.83	0.00	6	PM_10_ + PM_2.5_ pollution
Group 4	1.20	8.46	13.87	0.14	269	O_3_ + PM_2.5_ pollution
Mean	4.43	16.77	20.49	0.20	337	

**Table 4:** The goodness of fit among the OLS, GWR and MGWR models.

Goodness of Fit Statistic	OLS	GWR	MGWR
Residual sum of squares	168.862	34.963	35.712
Log likelihood	−362.321	−96.184	−99.764
AIC	752.642	388.233	358.079
AICc	756.133	469.288	407.464
R^2^	0.5	0.897	0.894
Adj. R^2^	0.48	0.855	0.862
BIC		762.635	661.153
Degree of Dependency (DoD)		0.668	0.704

**Table 5:** Comparison of regression parameters between OLS and MGWR models.

Variable	OLS Model	MGWR Model				
	Est.	Mean	STD	Min	Median	Max
Intercept	0.000	0.885	0.356	0.396	0.772	1.779
popden	−0.006	0.173	0.213	−0.055	0.068	0.596
pgdp	−0.173 ***	0.008	0.095	−0.320	0.019	0.166
prosec	−0.015	−0.030	0.023	−0.104	−0.021	−0.011
FDI	0.115 **	−0.040	0.004	−0.054	−0.039	−0.035
NDVI	−0.141 **	−0.204	0.011	−0.247	−0.203	−0.186
roadden	0.208 ***	−0.014	0.001	−0.022	−0.014	−0.011
energy	0.069	0.022	0.154	−0.392	0.013	0.417
tem	0.230 ***	−0.437	0.324	−0.833	−0.466	0.079
pre	−0.541 ***	0.015	0.183	−0.236	−0.036	0.361
ws	−0.149 **	0.056	0.223	−0.571	0.085	0.443
rh	−0.461 ***	−0.080	0.287	−0.810	−0.088	0.490
ap	0.435 ***	0.535	0.089	0.403	0.518	0.658
sd	−0.315 ***	−0.019	0.127	−0.271	0.050	0.103

Note: ** *p* < 0.05, *** *p* < 0.01.

## Data Availability

Not applicable.

## References

[1] (2020). China Statistical Yearbook 2020.

[2] Zhan D.S., Kwan M.P., Zhang W.Z., Yu X.F., Meng B., Liu Q.Q. (2018). The driving factors of air quality index in China. J. Clean. Prod..

[3] Wang S.J., Zhou C.S., Wang Z.B., Feng K.S., Hubacek K. (2017). The characteristics and drivers of fine particulate matter (PM_2.5_) distribution in China. J. Clean. Prod..

[4] World Bank Pollution. https://www.worldbank.org/en/topic/pollution#1.

[5] Liu B.M., Ma Y.Y., Gong W., Zhang M., Yang J. (2018). Study of continuous air pollution in winter over Wuhan based on ground-based and satellite observations. Atmos. Pollut. Res..

[6] Wang Y.S., Yao L., Wang L.L., Liu Z.R., Ji D.S., Tang G.Q., Zhang J.K., Sun Y., Hu B., Xin J.Y. (2014). Mechanism for the formation of the January 2013 heavy haze pollution episode over central and eastern China. Sci. China Earth Sci..

[7] Cai W.Y., Xu X.D., Cheng X.H., Wei F.Y., Qiu X.F., Zhu W.H. (2020). Impact of “blocking” structure in the troposphere on the wintertime persistent heavy air pollution in northern China. Sci. Total Environ..

[8] Hu C.-Y., Gao X., Fang Y., Jiang W., Huang K., Hua X.-G., Yang X.-J., Chen H.-B., Jiang Z.-X., Zhang X.-J. (2020). Human epidemiological evidence about the association between air pollution exposure and gestational diabetes mellitus: Systematic review and meta-analysis. Environ. Res..

[9] Chen Y., Ebenstein A., Greenstone M., Li H. (2013). Evidence on the impact of sustained exposure to air pollution on life expectancy from China’s Huai River policy. Proc. Natl. Acad. Sci. USA.

[10] Shen Y., Wu Y.Y., Chen G.D., Van Grinsven H.J.M., Wang X.F., Gu B.J., Lou X.M. (2017). Non-linear increase of respiratory diseases and their costs under severe air pollution. Environ. Pollut..

[11] Chen R., Zhao Z., Kan H. (2013). Heavy smog and hospital visits in Beijing, China. Am. J. Respir. Crit. Care Med..

[12] Bai L., Yang J., Zhang Y., Zhao D., Su H. (2020). Durational effect of particulate matter air pollution wave on hospital admissions for schizophrenia. Environ. Res..

[13] Xiao Q.Y., Geng G.N., Liang F.C., Wang X., Lv Z., Lei Y., Huang X.M., Zhang Q., Liu Y., He K.B. (2020). Changes in spatial patterns of PM2.5 pollution in China 2000-2018: Impact of clean air policies. Environ. Int..

[14] Wei G.E., Zhang Z.K., Ouyang X., Shen Y., Jiang S.N., Liu B.L., He B.J. (2021). Delineating the spatial-temporal variation of air pollution with urbanization in the Belt and Road Initiative area. Environ. Impact Assess. Rev..

[15] Ye W.F., Ma Z.Y., Ha X.Z., Yang H.C., Weng Z.X. (2018). Spatiotemporal patterns and spatial clustering characteristics of air quality in China: A city level analysis. Ecol. Indic..

[16] Ma G.Z., Hofmann E.T. (2019). Immigration and environment in the U.S.: A spatial study of air quality. Soc. Sci. J..

[17] Li J., Garshick E., Hart J.E., Li L.X., Shi L.H., Al-Hemoud A., Huang S.D., Koutrakis P. (2021). Estimation of ambient PM2.5 in Iraq and Kuwait from 2001 to 2018 using machine learning and remote sensing. Environ. Int..

[18] Jiang W., Gao W.D., Gao X.M., Ma M.C., Zhou M.M., Du K., Ma X. (2021). Spatio-temporal heterogeneity of air pollution and its key influencing factors in the Yellow River Economic Belt of China from 2014 to 2019. J. Environ. Manag..

[19] Xu L.J., Zhou J.X., Guo Y., Wu T.M., Chen T.T., Zhong Q.J., Yuan D., Chen P.Y., Ou C.Q. (2017). Spatiotemporal pattern of air quality index and its associated factors in 31 Chinese provincial capital cities. Air Qual. Atmos. Health.

[20] Shen Y., Zhang L.P., Fang X., Ji H.Y., Li X., Zhao Z.W. (2019). Spatiotemporal patterns of recent PM2.5 concentrations over typical urban agglomerations in China. Sci. Total Environ..

[21] Shen F.Z., Zhang L., Jiang L., Tang M.Q., Gai X.Y., Chen M.D., Ge X.L. (2020). Temporal variations of six ambient criteria air pollutants from 2015 to 2018, their spatial distributions, health risks and relationships with socioeconomic factors during 2018 in China. Environ. Int..

[22] Lin X.Q., Wang D. (2016). Spatiotemporal evolution of urban air quality and socioeconomic driving forces in China. J. Geogr. Sci..

[23] Yuan M., Huang Y.P., Shen H.F., Li T.W. (2018). Effects of urban form on haze pollution in China: Spatial regression analysis based on PM2.5 remote sensing data. Appl. Geogr..

[24] Luo J.Q., Du P.J., Samat A., Xia J.S., Che M.Q., Xue Z.H. (2017). Spatiotemporal Pattern of PM2.5 Concentrations in Mainland China and Analysis of Its Influencing Factors using Geographically Weighted Regression. Sci. Rep..

[25] Yang J.H., Ji Z.M., Kang S.C., Zhang Q.G., Chen X.T., Lee S.Y. (2019). Spatiotemporal variations of air pollutants in western China and their relationship to meteorological factors and emission sources. Environ. Pollut..

[26] Wang S.J., Liu X.P., Yang X., Zou B., Wang J.Y. (2018). Spatial variations of PM2.5 in Chinese cities for the joint impacts of human activities and natural conditions: A global and local regression perspective. J. Clean. Prod..

[27] Hao Y., Liu Y.M. (2016). The influential factors of urban PM2.5 concentrations in China: A spatial econometric analysis. J. Clean. Prod..

[28] Abdo A.-B., Li B., Zhang X., Lu J., Rasheed A. (2020). Influence of FDI on environmental pollution in selected Arab countries: A spatial econometric analysis perspective. Environ. Sci. Pollut. Res..

[29] Yu M.Y., Xu Y., Li J.Q., Lu X.C., Xing H.Q., Ma M.L. (2021). Geographic Detector-Based Spatiotemporal Variation and Influence Factors Analysis of PM2.5 in Shandong, China. Pol. J. Environ. Stud..

[30] Xu G.Y., Ren X.D., Xiong K.N., Li L.Q., Bi X.C., Wu Q.L. (2020). Analysis of the driving factors of PM2.5 concentration in the air: A case study of the Yangtze River Delta, China. Ecol. Indic..

[31] Liu H.M., Fang C.L., Zhang X.L., Wang Z.Y., Bao C., Li F.Z. (2017). The effect of natural and anthropogenic factors on haze pollution in Chinese cities: A spatial econometrics approach. J. Clean. Prod..

[32] Liu Q.Q., Wu R., Zhang W.Z., Li W., Wang S.J. (2020). The varying driving forces of PM2.5 concentrations in Chinese cities: Insights from a geographically and temporally weighted regression model. Environ. Int..

[33] Guo B., Wang X.X., Pei L., Su Y., Zhang D.M., Wang Y. (2021). Identifying the spatiotemporal dynamic of PM2.5 concentrations at multiple scales using geographically and temporally weighted regression model across China during 2015–2018. Sci. Total Environ..

[34] Zhan D., Kwan M.-P., Zhang W., Wang S., Yu J. (2017). Spatiotemporal Variations and Driving Factors of Air Pollution in China. Int. J. Environ. Res. Public Health.

[35] Zhou C.S., Li S.J., Wang S.J. (2018). Examining the Impacts of Urban Form on Air Pollution in Developing Countries: A Case Study of China’s Megacities. Int. J. Env. Res. Pubulic Health.

[36] Anselin L. (2002). Under the hood issues in the specification and interpretation of spatial regression models. Agric. Econ..

[37] Moore T.W., Dixon R.W. (2015). A Spatiotemporal Analysis and Description of Hurricane Ivan’s (2004) Tornado Clusters. Pap. Appl. Geogr..

[38] Brunsdon C., Fotheringham S., Charlton M. (1998). Geographically weighted regression. J. R. Stat. Soc. Ser. D.

[39] Fotheringham A.S., Yang W., Kang W. (2017). Multiscale geographically weighted regression (MGWR). Ann. Am. Assoc. Geogr..

[40] Wang X., Zhou D.Q. (2021). Spatial agglomeration and driving factors of environmental pollution: A spatial analysis. J. Clean. Prod..

[41] Jiang H.Y., Li H.R., Yang L.S., Li Y.H., Wang W.Y., Yan Y.C. (2014). Spatial and Seasonal Variations of the Air Pollution Index and a Driving Factors Analysis in China. J. Environ. Qual..

